# The RNA Chaperone Hfq Is Involved in Colony Morphology, Nutrient Utilization and Oxidative and Envelope Stress Response in *Vibrio alginolyticus*

**DOI:** 10.1371/journal.pone.0163689

**Published:** 2016-09-29

**Authors:** Yiqin Deng, Chang Chen, Zhe Zhao, Jingjing Zhao, Annick Jacq, Xiaochun Huang, Yiying Yang

**Affiliations:** 1 Key Laboratory of Tropical Marine Bio-resources and Ecology, South China Sea Institute of Oceanology, Chinese Academy of Sciences, Guangzhou, China; 2 Guangdong Provincial Key Laboratory of Applied Marine Biology, South China Sea Institute of Oceanology, Chinese Academy of Sciences, Guangzhou, China; 3 Xisha/Nansha Ocean observation and research station, South China Sea Institute of Oceanology, Chinese Academy of Sciences, Guangzhou, China; 4 University of Chinese Academy of Sciences, Beijing, China; 5 Institute for Integrative Biology of the Cell (I2BC), CEA, CNRS, Université Paris-Sud, Université Paris-Saclay, Gif-sur-Yvette, France; University of Helsinki, FINLAND

## Abstract

Hfq is a global regulator that is involved in environmental adaptation of bacteria and in pathogenicity. To gain insight into the role of Hfq in *Vibrio alginolyticus*, an *hfq* deletion mutant was constructed in *V*. *alginolyticus* ZJ-T strain and phenotypically characterized. Deletion of *hfq* led to an alteration of colony morphology and reduced extracellular polysaccharide production, a general impairment of growth in both rich medium and minimal media with different carbon sources or amino acids, enhanced sensitivity to oxidative stress and to several antibiotics. Furthermore, a differential transcriptomic analysis showed significant changes of transcript abundance for 306 protein coding genes, with 179 genes being up regulated and 127 down-regulated. Several of these changes could be related to the observed phenotypes of the mutant. Transcriptomic data also provided evidence for the induction of the extracytoplasmic stress response in absence of Hfq. Altogether, these findings point to broad regulatory functions for Hfq in *V*. *alginolyticus* cells, likely to underlie an important role in pathogenicity.

## Introduction

*Vibrio alginolyticus* is a common gram-negative halophilic bacterium that is part of the normal microflora in marine environment worldwide. However, more and more studies suggest that it is also a potential threat to marine animals and humans by causing serious infections [1–3]. A recent report showed that the incidence of diseases caused by vibrios in USA has dramatically increased in the last decade and *V*. *alginolyticus* has now been listed as one of the most common pathogens together with *Vibrio parahaemolyticus* and *Vibrio vulnificus* [4]. Indeed, *V*. *alginolyticus* was proposed to correspond to biotype 2 of *V*. *parahaemolyticus* since these two species have almost indistinguishable phenotypes except for the ability to utilize sucrose [5]. The risk of outbreaks caused by *V*. *alginolyticus* is underestimated compared with its close relatives. Its pathogenic mechanism, adaptation and epidemic traits remain unknown.

To respond to environmental cues, bacterial cells need to coordinate their gene expression quickly and precisely. Regulatory non-coding small RNAs (sRNAs) are now known to play essential roles in post-transcriptional regulation of gene expression and in virulence [6, 7]. Bacterial sRNAs can regulate target mRNAs positively or negatively, at the translational level or through affecting mRNA stability [8]. Many sRNAs exert their functions by incomplete base pairing with target mRNAs. Such interactions often require the assistance of the RNA chaperone Hfq, an RNA-binding protein belonging to the Sm protein family [8]. Hfq binds to both target mRNA and sRNA and stabilizes the interaction by a mechanism not fully understood [9]. In addition, in case of a negative regulation by the sRNA, Hfq may be involved in the recruitment of RNase E for further degradation of both the mRNA and the sRNA [8, 9]. More rarely, interaction of the sRNA with its target can interfere with RNase E cleavages and stabilize the mRNA (see [10] for a review).

Deletion of *hfq* was reported to affect motility, biofilm formation, central metabolism, virulence and stress response in a variety of species mostly gram-negative [11–14]. For instance, in *Salmonella*, the expression of at least 20% genes (about 1,000 genes) is affected by Hfq [12, 13]. Compared to other gram-negative species, especially *Escherichia coli* and *Salmonella sp*., still relatively little is known about the role of Hfq and sRNAs in *Vibrio sp*. In *V*. *cholerae*, sRNAs present in multiple copies, such as Qrr or CsrBs, the latters targetting the CsrA protein rather than mRNAs [15] have been found to be important regulators of the quorum sensing response in *V*. *cholerae* and *V*. *harveyi* [16–18], which itself controls virulence gene expression in pathogenic *Vibrio* [19, 20]. Hfq was also found to down regulate expression of the alternative extracytoplasmic stress (ECS) RpoE sigma factor in *V*. *cholerae* [21]. RpoE itself controls the production of the sRNA VrrA, which represses translation of the outer membrane protein OmpT in an Hfq dependent manner [22]. In *V*. *parahaemolyticus*, a *Δhfq* mutant is more resistant to oxidative stress and has increase production of the thermostable direct hemolysin (TdH), an important virulence factor in this species [23, 24].

In *V*. *alginolyticus*, a previous study showed that deletion of *hfq* decreased motility, biofilm formation and virulence [14]. In this study, we further characterized potential roles of Hfq in *V*. *alginolyticus* by phenotypic and transcriptomic analyses of the deletion mutant. Our data show that Hfq participates in diverse cellular processes including colony morphology, nutrient utilization, and envelope composition by modulating the expression of a large variety of genes some of which are likely to be controlled by Hfq-dependent sRNAs.

## Materials and Methods

### Bacterial strains, plasmids and growth conditions

The bacterial strains and plasmids used in this study are listed in Table 1.

**Table 1:** Strains and plasmids used in this study.

Strain or plasmid	Relevant characteristics	Source
***V*. *alginolyticus***		
ZJ-T	Ap^r^ (ampicillin resistant), translucent/smooth variant of wild strain ZJ51 [25]; isolated from diseased *Epinephelus coioides* off the Southern China coast	[26]
*Δhfq*-T	Ap^r^; ZJ-T carrying an in-frame deletion of *hfq* gene	This study
*hfq*^+^-T	Ap^r^, Cm^r^ (chloramphenicol resistant), *Δhfq*-T, z:: pNQ705-1-*hfq*	This study
***E*. *coli***		
SY327	*Δ*(*lac pro) argE*(Am) *rif malA recA56* λ*pir* lysogen; suicide vector pDM4’s intermediate host	[27]
S17-1	*thi pro hsdR hsdM*^*+*^*recA* RP4-2-Tc::Mu-Km::Tn*7* λ*pir* lysogen; donor strain for conjugation	[28]
**Plasmids**		
pDM4	Cm^r^; suicide vector with an R6K origin, requiring the Pir protein for its replication, and the *sacBR* gene of *Bacillus subtilis*	[29]
pNQ705-1	Cm^r^; suicide vector with a Pir dependent, R6K origin	[29]
pDM4-*Δhfq*	Cm^r^; pDM4 containing the mutant allele of *hfq*	This study
pNQ705-1-*hfq*	Cm^r^; pNQ705-1 containing the wild-type allele of *hfq*	This study

*V*. *alginolyticus* was cultured in Tryptic Soy Broth (TSB) (BD, USA) at 30°C. *Escherichia coli* strains were cultured in Luria–Bertani (LB) (Invitrogen, USA) medium supplemented with appropriate antibiotics at 37°C. For transconjugants selection, TCBS medium (BD, USA) was used with 5 μg.ml^-1^ chloramphenicol (Cm). To select transconjugants having undergone plasmid excision and allelic exchange, expression of the *sacB* gene carried by the pDM4 plasmid was induced by adding 10% sucrose to the medium. This gene encodes levansucrase which is lethal for gram-negative bacteria [29]. For colony morphology observation, LB agar plates were used. Unless otherwise indicated, M63 minimum medium was made according to the following recipe: 3 g.L^-1^ KH_2_PO_4_, 7 g.L^-1^ K_2_HPO_4_, 2 g.L^-1^ (NH_4_)_2_SO_4_, 0.5×10^−3^ g.L^-1^ FeSO_4_, 30 g.L^-1^ NaCl, 2×10^−3^ M MgSO_4_, 5×10^−3^ g.L^-1^ thiamine and 4 g.L^-1^ D-glucose.

### Construction of a *hfq* mutant and complementation strain

**Fig 1:**
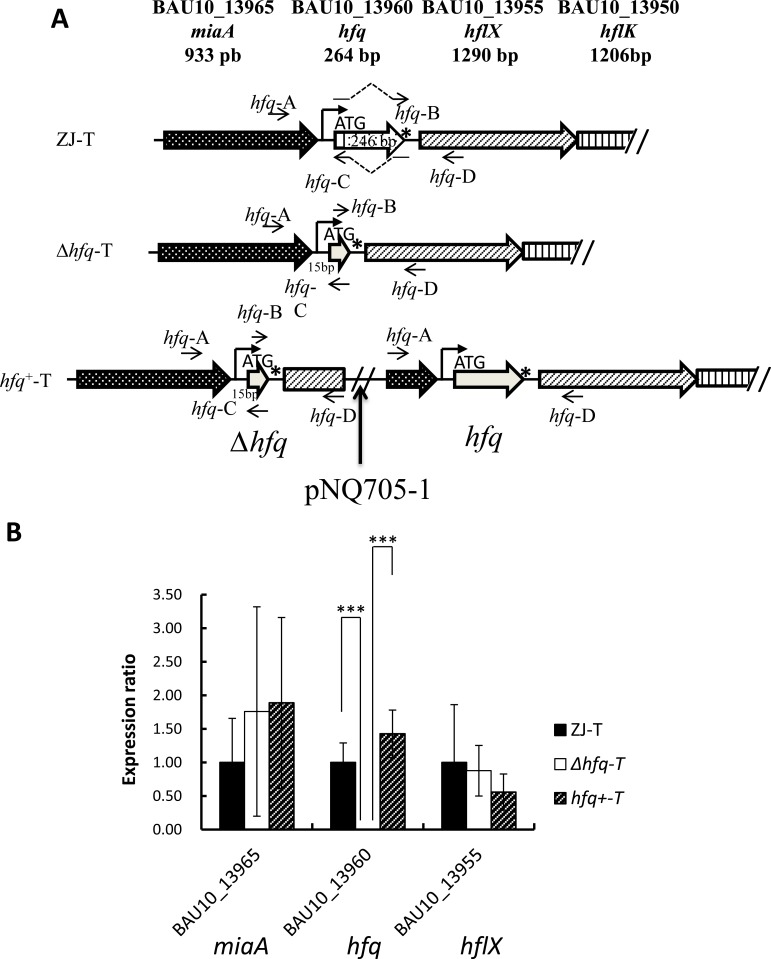
**Structure of the *hfq* locus (A) and neighboring gene expression in *Vibrio alginolyticus* ZJ-T and derivative strains (B). A.** The *hfq* gene is downstream of the *miaA* gene (locus ID = BAU10_13965) and upstream of 3 genes (*hflX*, *K*, *C*) likely to form an operon. A putative Sigma70 promoter identified upstream of *hfq* is indicated. 246 bps of the *hfq* ORF, from the 6th codon to the stop codon not included (marked by a star) were deleted as described in Materials and Methods, giving rise to the Δ*hfq*-T strain. This mutation does not affect the potential promoter of the operon, neither the ribosome binding site of the downstream gene. A wild type *hfq* gene was reinserted at the Δ*hfq* locus of the Δ*hfq*-T strain by insertion of pNQ705-1-*hfq* by homologous recombination (see Materials and Methods for details) generating a duplication of the locus, corresponding to the fragment inserted in pNQ705-1, from primer *hfq*-A to primer *hfq*-D. The depicted situation corresponds to an insertion of the plasmid downstream of the *hfq* deletion. **B**. Relative expression of *hfq*, the upstream gene (*miaA*) and the downstream gene (*hflX*) in derivative strains compared to WT. Relative expression (normalized to the WT level for each gene) was determined by qPCR as described in Materials and Methods. Error bars correspond to standard deviations from three biological replicates. Statistically significant differences are indicated (***p < 0.001).

An in-frame deletion of the *hfq* gene, retaining the five first codons of the gene (15 bp) and the stop codon was constructed in *V*. *alginolyticus* ZJ-T strain by overlapping extension (SOE) PCR as described by Milton *et al* [29]. Briefly, two fragments flanking the *hfq* deletion (Fig 1A), were amplified with two pairs of primers, *hfq*-A and -B and *hfq*-C and -D respectively, B and C containing overlapping extensions (S1 Table). These two fragments were further assembled by PCR with primer pair *hfq*-A and *hfq*-D, producing a fragment comprising the 321 bp upstream and 249 bp downstream regions of the *hfq* gene, and including. This fragment was then cloned into the suicide vector pDM4, generating plasmid pDM4-Δ*hfq* (Table 1), using *E*. *coli* SY327 as an intermediate host. The recombinant plasmid was transferred by conjugation from strain S17-1 (Table 1) to *V*. *alginolyticus* wild-type strain ZJ-T before allelic exchange as described above. The presence of the *hfq* in-frame deletion was then confirmed by sequencing and the strain named Δ*hfq*-T (Fig 1A, Table 1).

A complementation strain, carrying both the deleted allele and the WT allele, was constructed as described previously [30–32]. Briefly, a PCR fragment containing the *hfq* gene and its flanking regions (including the *hfq* gene native promoter, *hfq* being the first gene of an operon -see Fig 1A) was amplified using the primers *hfq*-A and *hfq*-D. The fragment was then inserted into plasmid pNQ705-1 and the recombinant plasmid, pNQ705-1-*hfq*, was transferred into the *hfq* mutant by conjugation, leading to integration of pNQ705-1-*hfq* at the *hfq* locus by homologous recombination. The resulting strain has two copies of the *hfq* locus, one mutant, one WT. The presence of an intact *hfq* gene was confirmed by PCR analysis and sequencing. The complementation strain was named *hfq*^+^-T. (Fig 1A, Table 1). Quantitative RT-PCR (qPCR) was used to test a potential effect of the *hfq* deletion on neighboring gene expression (potential polar effects) as described below.

### Quantitative RT-PCR

Overnight cell cultures of ZJ-T, Δ*hfq*-T and *hfq*^*+*^-T were diluted to OD_600_ = 0.01 in TSB medium and grown to early exponential phase (OD_600_ = 0.5) and bacterial cells were collected. All reagents were from Takara Bio Inc. Total RNA was isolated by using RNAiso Plus, DNase-treated and 1 μg of RNA was reverse-transcribed with PrimeScript^TM^ RT reagent Kit. PCR amplification was then carried out on a RotorGene RG-3000 (Qiagen) using SYBR Premix Ex Taq™ with primers specific to the genes to be assayed (S1 Table). Three housekeeping genes (*recA*, *uvrA* and *gyrA*) were used as internal controls. Relative gene expression was calculated by the 2^−ΔΔCt^ method [33] and normalized to the wild type ZJ-T value. Measurements were done in triplicates. Statistical significance was assayed by one-way ANOVA LDS method (*p < 0.05, **p < 0.01, ***p < 0.001).

### Colony and cell morphology

To observe colony morphology, overnight cell cultures were serially diluted and 100 μl of each dilution was spread on LB agar plates. Single colonies were observed using the BIO-RAD image system Gel Doc^TM^ XR+. Bacterial cell morphology was observed by Transmission Electron Microscopy (Hitachi H-7650 TEM). Briefly, ZJ-T, Δ*hfq*-T and *hfq+*-T strains were cultured for 8–10 hours in TSB, then placed on copper grids and negatively stained with 3% phosphotungstic acid at pH 7.0 for 2 min.

### Extracellular polysaccharide (EPS) quantification

To quantify EPS, alcian blue which stains acidic polysaccharides was used as described by Antony Croxatto *et al* [34] with modification. Briefly, 5 μl overnight cultures of ZJ-T, Δ*hfq*-T and *hfq*+-T were spotted on LB agar plate and incubated at 30°C for 24 h. Bacterial spots were scraped from the plate and re-suspended in filtered 4 mM EDTA to a same final OD_600_ = 1.0. EPS was sheared off the cells by three successive centrifugations at 25,000 g for 30 min followed by re-suspension in the same volume of 4mM EDTA. 1 ml of the supernatant was mixed with 4 ml alcian blue solution and incubated at room temperature for one hour. The solution was centrifuged at 1,500 g for 20 min, and the supernatant was discarded carefully. The pellet was washed in 4 ml ethanol once and re-suspended in 4 ml 10% SDS. The absorbance at 620 nm was recorded. The average quantity of EPS was normalized as OD_620/_OD_600_ and one-way ANOVA LDS method was used for statistical analysis of the difference between the wild type, the mutant and the complementation strain with IBM SPSS Statistics 19. *p* <0.05 was retained as the threshold for statistical significance.

### Measurement of bacterial growth

Bacterial strains were grown overnight in TSB medium at 30°C with shaking at 200 rpm. To test growth in rich medium, each culture was brought to OD_600_ = 1.0 using fresh TSB and then diluted into fresh TSB (1:1000). To investigate the effect of various carbon sources on growth, M63 was modified by replacing D-glucose with 0.4% (w/v) of different carbohydrates, (D-maltose, D-trehalose, D-fructose, *N*-acetyl-D-glucosamine) or TCA cycle intermediates (sodium citrate, α-ketoglutaric acid, sodium succinate, sodium malonate, oxaloacetic acid and fumaric acid). To probe the effect of amino acid(s) on growth, D-glucose and (NH_4_)_2_SO_4_ in M63 were left out and replaced by the nonpolar amino acids L-alanine (150mM), L-valine (20mM), L-leucine (20mM) or L-isoleucine (20mM), and polar amino acids L-threonine (50mM), L-aspartic acid (10mM), L-lysine (15mM), L-glutamic acid (10mM), L-serine (50mM), L-arginine (50mM), L-histidine hydrochloride (50mM) or L-glutamine (10mM) as both carbon and nitrogen source. Minimal medium assays were carried out as follow: Overnight cultures were collected by centrifugation and washed once with M63 without D-glucose and (NH_4_)_2_SO_4_, then individually re-suspended in the above modified M63 to the same cell density. Cultures (3 replicates in each case) were then incubated at 30°C with continuous shaking at 200 rpm in 96 well plates. OD_600_ was measured at regular time intervals using the Multiskan Ascent plate reader (Thermo Fisher Scientific).

### Stress response assays

Briefly, overnight cultures in TSB of ZJ-T, Δ*hfq*-T and *hfq*+-T were re-suspended at OD_600_ = 5.0 (2.5×10^9^ CFU/ml), and then serially diluted 10 fold into fresh TSB medium. 5 μl of each dilution was spotted on TSB agar plates alone or containing 6mM CuSO_4_ or 0.0015% H_2_O_2_ and incubated at 30°C for 24 h.

### Antibiotic susceptibility assays

Antibiotics were bought from Hangzhou Binge Microorganism Reagent Co., and disk diffusion assays were carried out as described previously [35] with modifications. Disks with various concentrations of antibiotics were made. 200 μl of overnight bacterial culture was added to 10 ml fresh TSB medium, then mixed gently and plated on TSB agar plates. Disks were placed on the dried plates and incubated at 30°C for 24 h before measuring the inhibition zone. Measurements were done in triplicates. Statistical significance was assayed by one-way ANOVA LDS method as described above (*p* < 0.05).

### Transcriptome sequencing and bioinformatic analysis

Two single colonies from strains ZJ-T and Δ*hfq*-T were used to inoculate two independent cultures in TSB medium, which were grown at 30°C to early exponential phase (OD_600_ = 0.5). Equal amount of cells were harvested from each sample and total RNA was isolated using RNAiso Plus (Takara Bio Inc.) according to the manufacturer's instructions. The quality and quantity of total RNA was verified by measuring the OD_260_/_280_ and OD_260_/_230_ with a Nanodrop 2000 spectrophotometer, and agarose gel electrophoresis. The whole process was repeated once and four RNA samples from each strain were pooled for RNA-seq analysis. Libraries derived from the mixed RNA samples were sequenced on an Illumina HiSeq^TM^ 2000 at the Beijing Genomics Institute using standard Illumina protocols.

Primary sequencing data produced by Illumina HiSeq^TM^ 2000 were subjected to quality control. Filtered quality reads were mapped to the reference genome sequence of *V*. *alginolyticus* ZJ-T (GenBank accession numbers CP016224 and CP016225 for Chromosome 1 and 2 respectively) using the SOAP2 aligner [36]. To identify differentially expressed transcripts, the mapped sequence reads were normalized as Reads Per Kilobase per Million (RPKM) for each annotated feature in the genome as described previously [37] and the expression ratio (fold change) between mutant and wild type was computed. The significance of differential gene expression (*p*-value) was then calculated by the method of Audic and Claverie [38]. In addition a False Discovery Rate (*FDR*) was calculated by the method of Benjamini and Yekutieli [39]. Differentially expressed genes (expression ratio ≥2, *p*-value <0.1 and *FDR <* 0.001) were classified into different categories based on Kyoto Encyclopedia of Genes and Genomes (KEGG) pathways and ontologies. Raw sequence reads were deposited in the SRA databank under the accession number SRP076968.

### Outer membrane protein analysis

Extraction of total outer membrane proteins (OMPs) was conducted as previously described [40]. Briefly, overnight cultures in TSB of ZJ-T, Δ*hfq*-T and *hfq+*-T were diluted to OD_600_ = 0.01 in 15 ml TSB medium and incubated at 30°C to OD_600_ = 0.5. The cultures were centrifuged at 1,000 g for 10 min and the pellets were re-suspended in 13.5 ml ddH2O. The cells were broken by sonication followed by cell debris removal by centrifugation at 12,000 g for 10 min at 4°C. The supernatants were transferred to new tubes containing 1.5 ml of 20% sodium sarcosine, to solubilize inner membrane proteins without affecting OMPs. After incubation at room temperature for 30 min, followed by centrifugation at 240,000 g for 1 h at 4°C in an Beckman Coulter Optima XPN-100 Ultracentrifuge, the pellets were washed with ice-cold ddH2O and re-centrifuged in the same condition before being re-suspended in 1×SDS sample buffer. 15 μl of protein preparation was separated by SDS–PAGE (12%). The gel was stained with Coomassie brilliant blue and photographed with a gelDoc imager (Bio-Rad, USA).

## Results

### Construction of an *hfq* null mutant and complementation

An in-frame deletion of the *hfq* gene, from the sixth codon to the last codon (not including the stop codon) was constructed as described in Materials and Methods. In addition, we constructed a complementation strain, where the WT *hfq* gene was reintroduced in the chromosome, generating a partial tandem duplication of the locus, containing both the deleted and the WT version of the gene (Fig 1A). Since *hfq* is the first gene of a four-gene operon, it was important to verify that neither the deletion nor the reintroduction of the wild-type gene had a polar effect on the neighboring genes. Hence, we checked by qPCR expression of all three genes (upstream, *hfq* and downstream gene) in the three strains: WT, Δ*hfq* and the complementation strain. As shown in Fig 1B, a significant difference of expression was observed only in the case of *hfq*: expectedly expression could not be detected in the mutant and was restored in the complementation strain.

### Hfq regulates colony morphology and synthesis of EPS in *V*. *alginolyticus*

**Fig 2:**
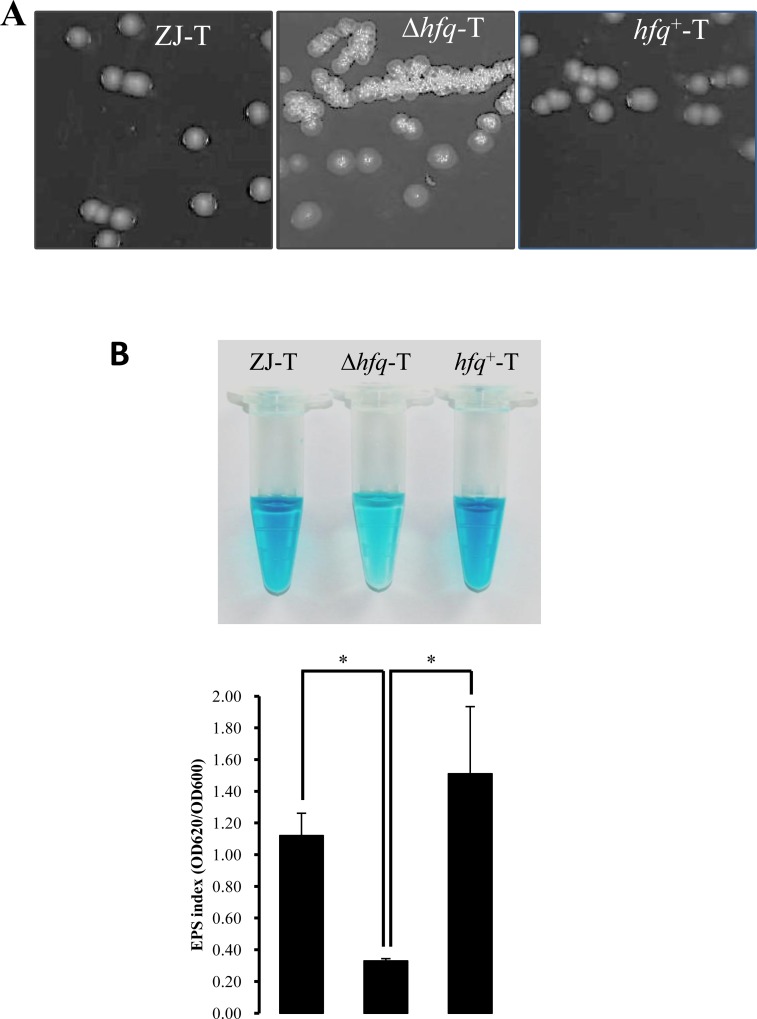
*hfq* deletion affects colony morphology and extracellular polysaccharide production. **A.** Colony morphology was observed with a BIO-RAD Gel DocTM XR+ imager. *hfq* deletion led to a colony morphology change from translucent and smooth to opaque and rugose. **B.** Amounts of extracellular polysaccharides of ZJ-T, Δ*hfq*-T and *hfq*+-T were assayed in triplicates with alcian blue (top) and quantified by spectroscopy (bottom). Error bars correspond to standard deviations. Statistically significant differences are indicated (* *p* < 0.05)

*V*. *alginolyticus* wild type ZJ-T strain forms translucent (Tr) and smooth (S) colonies on LB agar plates [26]. In contrast, we observed that the *hfq* mutant forms opaque (Op) and rugose (R) colonies and that reintroducing the WT *hfq* allele in strain *hfq*+-T reverted colony morphology to Tr-S (Fig 2A), indicating a role of Hfq in the morphology change. In *V*. *cholerae*, colony morphology changes from Tr to Op have been associated with changes in production of extracellular polysaccharides (Vps) [41, 42]. In *Vibrio vulnificus*, expression of a Group 1 capsular polysaccharide (CPS) operon was positively associated with an Op phenotype [43]. Whereas the transition from Tr to Op requires increased CPS production, transition from S to R does not [41, 44]. *V*. *vulnificus* carries another exopolysaccharide gene cluster, named *brp*, which was found to be important for rugose colony formation [44]. We quantified by alcian blue the EPS produced by cells grown on LB agar plates. As shown in Fig 2B, EPS staining by alcian blue was significantly decreased in the *hfq* mutant, compared to the wild type and the complementation strain, whereas transmission electron microscopy revealed no visible change in cell shape or size: all three strains carried similar 3–4 polar flagella and were surrounded by a thick layer of extracellular matrix (S1 Fig).

### Deletion of *hfq* affects carbon and nitrogen metabolism

When the bacteria grew both in TSB medium and in minimal medium supplemented with D-glucose, D-maltose, D-trehalose, D-fructose or *N*-acetyl-D-glucosamine as sole carbon source, the lag phase of Δ*hfq*-T was 2 hours longer than that of ZJ-T, and the growth rate of the mutant decreased in exponential phase, as well as total growth. When intermediates of the TCA cycle were supplied as sole carbon source, no differences between WT and mutant were observed in terms of duration of lag phase, but the *hfq* mutant displayed a significant decrease in growth yield. These phenotypes were reverted in the complementation strain (S2 Fig, Table 2). In addition, none of the strains were able to grow on sodium malonate (S2 Fig, Table 2).

**Table 2:** Effect of *hfq* deletion on growth rate and total growth in rich medium and minimal medium M63 supplemented with various carbon sources. Values correspond to the mean of three independent cultures. Significant statistical differences between strains and WT are indicated (*p < 0.05, **p < 0.01, ***p <0.001). Growth rates are expressed as generation times.

Medium	Generation time (minutes)			Total growth (OD_600_)		
	ZJ-T	Δ*hfq*-T	*hfq*+-T	ZJ-T	Δ*hfq*-T	*hfq*+-T
TSB	101.15±3.14	160.47±1.80 (***)^a^	90.70±3.16	1.27±0.02	1.15±0.02 (***)	1.28±0.01
M63+ d-glucose	144.46±3.17	195.71±5.24 (***)	120.19±9.20	0.53±0.02	0.45±0.01 (***)	0.53±0.00
M63+d-maltose	154.81±6.97	228.33±8.46 (***)	126.85±6.76	0.56±0.01	0.49±0.00 (***)	0.57±0.01
M63+d-trehalose	156.55±8.98	283.61±10.06 (***)	128.33±7.86	0.56±0.00	0.48±0.00 (***)	0.58±0.00
M63+d-fructose	119.48±5.44	160.42±7.64 (***)	101.79±8.96	0.53±0.00	0.45±0.01 (***)	0.54±0.00
M63+						
*N*-acetyl-d-glucosamine	145.79±7.29	188.58±5.25 (***)	123.97±9.91	0.5±0.00	0.40±0.00 (***)	0.49±0.00
Sodium citrate	459.47±40.41	881.25±50.39 (***)	418.03±49.48	0.29±0.02	0.21±0.00 (***)	0.29±0.03
α-ketoglutaric acid	118.37±2.28	189.04±5.73 (***)	94.59±6.08	0.47±0.00	0.38±0.00 (***)	0.47±0.00
Sodium succinate	162.74±8.19	156.12±7.60	137.71±3.75	0.25±0.00	0.21±0.00 (***)	0.25±0.00
Sodium malonate	-	-	-	-	-	-
Oxaloacetic acid	91.18±0.67	86.65±2.41	71.24±0.59	0.24±0.00	0.21±0.00 (***)	0.24±0.00
Fumaric acid	176.60±4.54	144.38±2.26 (***)	150.06±4.40	0.42±0.00	0.38±0.00 (***)	0.43±0.00

^**a**^ *p < 0.05

**p < 0.01

***p <0.001.

To check the impact of *hfq* on the metabolism of amino acids, bacterial growth was measured in M63 with no added (NH_4_)_2_SO_4_ or D-glucose and supplemented with individual amino acids as both carbon and nitrogen sources. As shown in S3 Fig and in Table 3, deletion of *hfq* affected the ability of *V*. *alginolyticus* to grow on L-threonine or L-arginine, which were the preferred amino acids for the wild type strain, suggesting that *hfq* is essential for the utilization of these amino acids. In addition, absence of *hfq* affected cell growth on L-alanine, L-serine, L-histidine or L-leucine (S3 Fig, Table 3). Other tested amino acids could not support growth of either wild type or mutant strains (S3 Fig, Table 3).

**Table 3:** Effect of *hfq* deletion on growth rates and total growth in minimal medium M63 supplemented with various amino acids as both carbon and nitrogen sources. Values correspond to the mean of three independent cultures. Significant statistical differences between strains and WT are indicated (*p < 0.05, **p < 0.01, ***p <0.001).

Amino acid	Generation time (minutes)			Total growth (OD_600_)		
	ZJ-T	Δ*hfq*-T	*hfq*+-T	ZJ-T	Δ*hfq*-T	*hfq*+-T
L-alanine	138.77±12.22	208.42±10.22 (***)^a^	110.72±2.93	0.57±0.01	0.41±0.01 (***)	0.59±0.01
L-threonine	249.71±10.75	- (***)	200.69±14.13	0.50±0.01	-(***)	0.53±0.01
L-aspartic acid	-	-	-	-	-	-
L-lysine	-	-	-	-	-	-
L-glutamic acid	-	-	-	-	-	-
L-serine	201.47±10.91	617.52±28.50 (***)	170.73±2.43	0.39±0.01	0.10±0.01 (***)	0.39±0.01
L-arginine	1616.96±93.16	- (***)	1686.25±165.43	0.29±0.05	- (***)	0.25±0.03
L-histidine hydrochloride	364.42±12.62	358.56±5.12	335.16±9.80	0.81±0.01	0.69±0.00 (***)	0.82±0.01
L-glutamine	-	-	-	-	-	-
L-leucine	1578.13±146.45	3727.20±263.49 (***)	1595.48±59.43	0.17±0.00	0.06±0.00 (***)	0.20±0.04
L-valine	-	-	-	-	-	-
L-isoleucine	-	-	-	-	-	-

^**a**^ *p < 0.05

**p < 0.01

***p <0.001.

### Deletion of *hfq* increased susceptibility of *V*. *alginolyticus* to copper and radical oxygen

**Fig 3:**
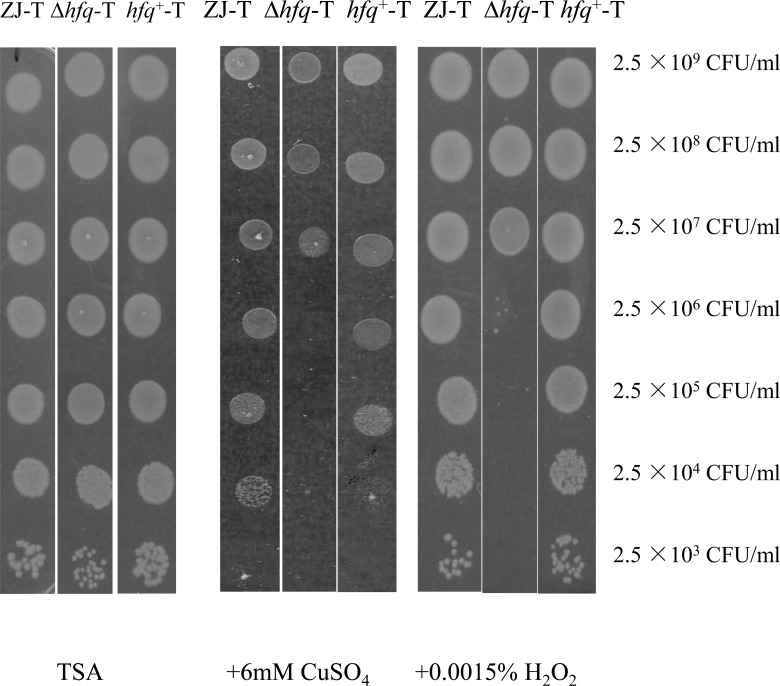
Δ*hfq*-T showed increased sensitivity to CuSO_4_ and H_2_O_2_. Serial dilution of cultures of ZJ-T, Δ*hfq*-T and *hfq*+-T were spotted on TSB agar plates (left panel), in presence of 6 mM CuSO4 (middle panel) or 0.0015% H_2_O_2_ (right panel). The picture is representative of the results of at least ten experiments.

Excessive amount of cupric ions are toxic to bacterial cells. Therefore, bacteria, including *V*. *alginolyticus*, have developed complex mechanisms to respond to this stressor [45, 46]. As shown in Fig 3, we observed a 1000 fold difference in survival rates between the *hfq* mutant and the WT on TSB agar plate in the presence of 6mM CuSO_4_. Excess of copper is toxic because cupric ions catalyze the formation of highly reactive hydroxyl radicals, generating a strong oxidative stress as does hydrogen peroxide. We found that the *hfq* mutant was also more sensitive to 0.0015% H_2_O_2_ (Fig 3)_._ Our results indicate that *hfq* plays a vital role in responding to oxidative stress generated by radical oxygen.

### Deletion of *hfq* altered antibiotic resistance of *V*. *alginolyticus*

Twenty-five antibiotic agents were used to determine the impact of *hfq* on antibiotic resistance. As shown in Table 4, all the strains show similar levels of resistance to lincomycin, clindamycin, ampicillin, oxacillin, piperacillin, penicillin-G, acetylspiramycin and amoxicillin. However, Δ*hfq*-T strain was more sensitive, compared to the wild type and the complemented strains, to vancomycin and fosfomycin, both of which inhibit the synthesis of cell wall, and kanamycin, tobramycin as well as neomycin, which belong to the aminoglycoside family of antibiotics and inhibit protein synthesis.

**Table 4:** Antibiotic resistance of ZJ-T, △*hfq*-T and *hfq*+-T.

Antibiotics	Concentration	Size of inhibition zone (mm)			*p*-value
	(μg/per disk)	ZJ-T	Δ*hfq*-T	*hfq*+-T	(Δ*hfq*-T *vs* ZJ-T) ^a^
lincomycin	2	0	0	0	1.00
**vancomycin**	**30**	**8.80±1.30**	**13.40±0.89**	**8.40±1.52**	**0.00**
clindamycin	2	0	0	0	1.00
streptomycin	300	12.60±1.52	14.80±1.92	12.67±2.08	0.24
ampicillin	10	0	0	0	1.00
oxacillin	1	0	0	0	1.00
piperacillin	100	0	0	0	1.00
**kanamycin**	**30**	**11.80±0.84**	**14.40±1.52**	**11.40±1.14**	**0.00**
penicillin-G	10	0	0	0	1.00
cefazolin	30	13.50±3.54	14.50±3.54	14.50±3.54	0.796
**tobramycin**	**10**	**11.00±2.00**	**14.67±0.58**	**11.00±1.73**	**0.03**
**neomycin**	**30**	**14.67±1.15**	**17.00±1.00**	**14.67±1.15**	**0.04**
novobiocin	30	19.00±3.46	21.00±3.61	18.33±2.31	0.34
erythromycin	15	16.00±1.00	17.67±3.06	16.33±1.53	0.36
medemycin	30	11.00±1.41	11.50±0.71	11.00±1.41	0.71
polymyxin B	300	13.50±2.12	14.00±1.41	12.50±2.12	0.81
acetylspiramycin	30	0	0	0	1.00
spectinomycin	100	12.50±0.71	11.00±1.41	10.50±2.12	0.40
cefixime	5	16.67±4.73	19.35±1.15	18.33±4.04	0.41
amoxicillin	10	0	0	0	1.00
azithromycin	15	17.00±1.00	19.67±2.52	18.00±1.00	0.11
clarithromycin	15	13.00±2.00	13.00±2.83	11.50±3.54	1.00
teicoplanin	30	8.50±0.71	10.00±0.00	8.50±0.71	0.08
**fosfomycin**	**200**	**17.00±1.73**	**22.67±0.58**	**15.00±1.00**	**0.00**
gentamicin	120	19.50±0.71	18.50±0.71	16.50±0.71	0.25

^**a**^ There is no significant difference (*p*>0.05) between the wild type ZJ-T and the complemented strain *hfq*+-T.

### Global effects of Hfq on *V*. *alginolyticus* transcriptome

Transcriptome sequencing of both the WT and the *Δhfq* strain by Illumina in early exponential phase (see Materials and Methods) was used to identify genes potentially regulated by *hfq* in *V*. *alginolyticus*. Of the 4710 protein coding genes annotated in the *V*. *alginolyticus* ZJ-T strain genome, 306 genes (approximately 6.5%) had their expression significantly affected in the mutant with our criteria (fold change ≥ 2, *p*-value ≤ 0.1 and a *FDR* ≤ 0.001) (S2 Table). Amongst them, 226 genes could be assigned to 24 KEGG functional categories based on their annotations.

Since Hfq typically regulates targets via the action of sRNAs, and that in some cases, interaction between the sRNA, Hfq and its target leads to degradation of both the target and the sRNA, one could expect some sRNA levels to be affected by the absence of Hfq. Accordingly, we identified 16 *V*. *alginolyticus* known sRNAs by sequence comparison with other vibrios and the sRNA database Rfam (http://rfam.xfam.org/)(S3 Table), and determine their expression levels in our transcriptomes. None of them filled out our criteria of statistical significance to be included in the list of differentially expressed genes, although some of them did show changes of expression, such as the *qrr*2-5 genes whose expression was undetectable in the *hfq* mutant (S3 Table).

Several categories of differentially expressed genes could be related to the phenotypes described in this study and are discussed below.

(1) Carbon uptake and central metabolism. As shown in S2 Table, 55 out of the 226 genes with predicted functions are involved in carbon utilization and energy conversion (Carbohydrate metabolism, Other carbon source utilization, Central and energy metabolism, and Carbon transport). Interestingly, the relative transcript abundances of the PTS system components II for trehalose, fructose, sucrose and mannitol were all decreased in the *hfq* mutant. In addition, genes involved in maltodextrin uptake (*malGEFK*) and conversion (*malQ*, *glgP*, *glgX*) as well as the regulatory gene *malT* were down regulated by 2–7 fold. Finally, the glycerol uptake facilitator genes *glpF* and *glpK* were also down regulated.

Conversely, many genes involved in glycolysis, TCA cycle and electron transport chains were up regulated in the *hfq* mutant. As shown in S2 Table, phosphoenolpyruvate carboxykinase (PckA) and phosphoenolpyruvate synthase (Pps), which convert oxaloacetate and pyruvate respectively to phosphoenolpyruvate, increased, whereas pyruvate kinase (Pyk) that catalyzes the reverse reaction of Pps was down regulated in the mutant. Other TCA cycle enzyme-encoding genes whose expression increased were *fumA*, *fumC*, *sucD* and *sucC*.

(2) Amino acids transport and metabolism. Growth of the *hfq* mutant on several amino acids was strongly affected compared to the WT (S3 Fig, Table 3). Our transcriptomic data suggest an increase of the biosynthesis pathways for glutamate in the mutant. Both genes for glutamate synthase (*gltD* and *gltB*), which synthesizes glutamate from glutamine, were up regulated in the *hfq* deletion background, and gamma-glutamyltranspeptidase (encoded by *ggt*), which converts glutathione to glutamate, was up regulated 3.7 fold. The valine/leucine/isoleucine biosynthetic pathway was also enhanced with an increase of 3-isopropylmalate dehydrogenase (*leuBC*) and ketol-acid reductoisomerase (*ilvC*). Conversely, genes such as *fadB*, *puuB* and *ilvD*, involved in valine/leucine/isoleucine degradation were down regulated.

Three genes encoding polar amino acid transporters of the general L-amino acid permease family (ABC transporters) showed decreased expression in the mutant. In *Rhizobium leguminosarum*, such ABC transporter, named Aap, has been shown to have a broad amino-acid specificity, importing not only polar amino acids such as glutamate, aspartate or histidine, but also more hydrophobic amino acids such as leucine and methionine [47]. *V*. *alginolyticus* encodes two such polar amino acid permeases, the two corresponding ATPase subunits being less expressed in the *hfq* mutant (*aapP1* and *aapP2*) as well as one polar amino acid transport system substrate-binding protein. Interestingly, the Aap transporter could also export its substrates, for instance glutamate [47].

(3) Motility and chemotaxis. Motility is driven by polar flagella in most *Vibrio* species in liquid environment and is required for infection [48, 49]. In *V*. *alginolyticus*, *hfq* was found to be required for motility [14], although we could not detect any obvious defect in flagella by TEM (S1 Fig). In contrast, our transcriptomic study provided a molecular basis for this phenotype since, as shown in S2 Table, 11 genes involved in motility or chemotaxis were down regulated by at least two fold, such as the four *fliC* genes, that encode flagellin. In addition, two motor proteins MotA and MotB that provide driving forces for motility were down regulated in the mutant as well as three of the four methyl-accepting chemotaxis proteins (MCP) and the MCP methyltransferase CheB/CheR fusion protein [49].

Finally, in *Vibrio* species, four to five sRNAs according to species, named Qrrs, are important mediators of Quorum sensing. At low cell density, Qrrs are made and block the translation of the master regulator gene *hapR* mRNA, in an Hfq dependent manner [16]. At high cell density, no Qrrs are made leading to expression of HapR. HapR is both a negative and positive transcriptional regulator and controls a number of genes, varying according to species, which are responding to the QS pathway [17]. We identified five Qrrs in *V*. *alginolyticus*, which showed very low expression in our transcriptomes (S3 Table). However, we could not detect any expression of Qrr2-4 in the *Δhfq* mutant. Consistent with this, we found that the *hapR* gene (BAU10_12125) was de-repressed 2.6 fold in the mutant.

### Hfq regulates OMP synthesis in *V*. *alginolyticus*

**Fig 4:**
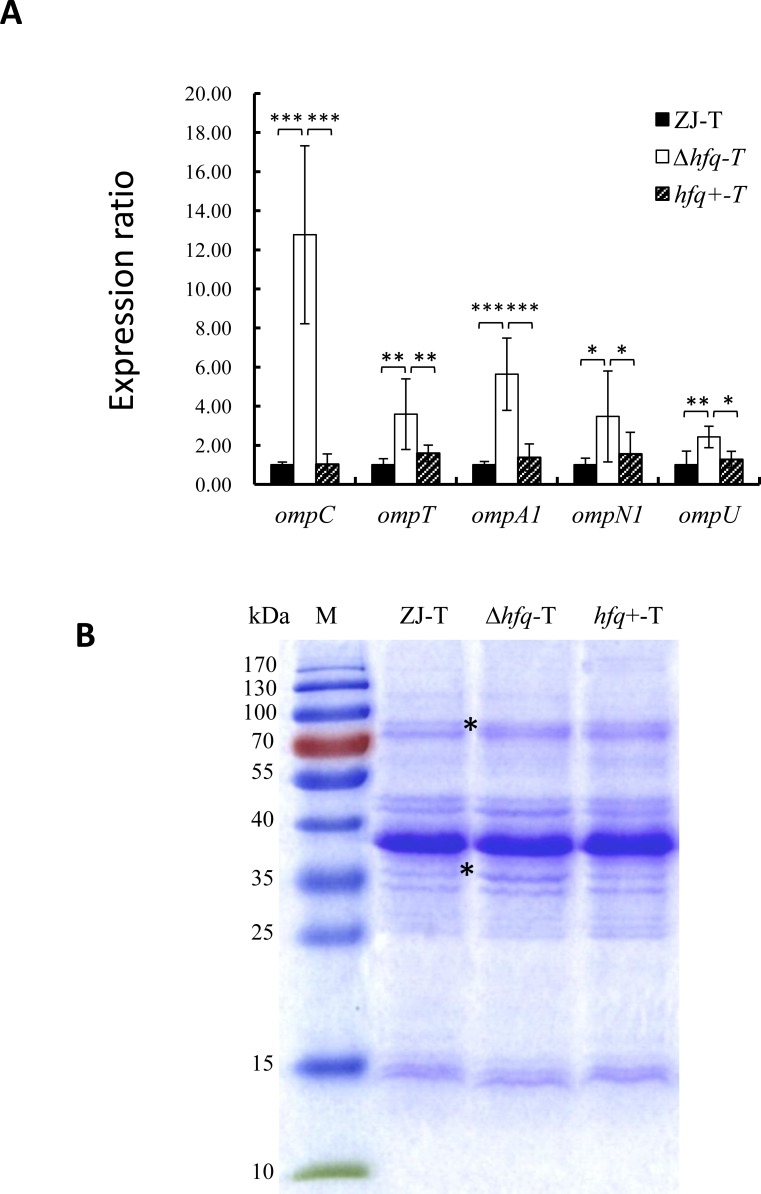
Hfq regulates outer membrane protein expression. **A.** Relative expression of OMP genes in derivative strains compared to WT. Relative expression (normalized to the WT level for each gene) was determined by qPCR as described in Materials and Methods. Error bars correspond to standard deviations from three biological replicates. Statistically significant differences are indicated (*p < 0.05, **p < 0.01, ***p <0.001). **B**. SDS-PAGE analysis of the outer membrane fraction from ZJ-T, *Δhfq*-T and *hfq*^+^-T. M: Molecular size markers. Apparent sizes of markers are indicated on the left. The positions of two bands that increase in the *hfq* mutant are indicated by stars.

Most outer membrane β-barrel proteins (OMPs) including OmpC, OmpT, OmpA, OmpN and OmpU were significantly up regulated in the *hfq* mutant, whereas second copies of *ompN* and *ompA* were down regulated (S2 Table). Indeed, *ompC* was the second most up regulated (65.8 X) gene in our data set, (although its expression level in the WT was 2000 fold less than this of *ompN1/ ompN2* together) and *ompT* was the fourth. This up-regulation of several OMP genes was further confirmed by qPCR (Fig 4A) and suggests that the outer membrane composition of *V*. *alginolyticus* is remodeled in absence of *hfq*. To further test this possibility, OMPs were extracted from strains ZJ-T, *Δhfq*-T and *hfq*+-T and were analyzed by SDS-PAGE. As shown in Fig 4B, a major band was observed at around the 38 kDa position, consistent with the high level of expression (data not shown) and the sizes of OmpN1 and OmpN2. The main difference between the WT and the mutant was the increase of a minor band corresponding to a size around 35 kDa, that could correspond to the less expressed OmpC. Finally, another minor band also increased in the 75 kDa region, which could correspond to one or several outer-membrane-localized iron receptor proteins in this size range (BAU10_23175, BAU10_12590, BAU10_23170), which were up regulated from 4 to 10 fold in our study (S2 Table).

## Discussion

It has been reported that approximately 50% of bacterial genomes carry homologs of *hfq* [50]. As an RNA molecular chaperone, Hfq exerts its function at the post-transcriptional level mainly by being required for the action of up to hundreds of regulatory non-coding sRNAs, the majority of them still uncharacterized. Hence, loss of Hfq often results in pleiotropic phenotypes in many bacteria [12, 13, 35, 51–55], vibrios not being exceptions [14, 21, 23]. In *V*. *alginolyticus*, Hfq had been previously shown to be required for motility, biofilm formation and resistance to various stress such as osmotic stress, ethanol and iron starvation [14]. The mutant was also found to be attenuated in virulence to zebra fish [14]. In here, we describe some additional phenotypes of the *hfq* mutant: colony morphology changes (Fig 2A), decrease in EPS production (Fig 2B), impaired growth on a range of carbon and nitrogen sources (Tables 2 and 3) and increased sensitivity to copper and hydrogen peroxide (Fig 3). In addition, a differential transcriptomic study showed that deletion of *hfq* led to altered expression of more than three hundred genes, with several of them that could be related to some of the phenotypes of the mutant.

A first obvious phenotype caused by *hfq* deletion is the colony appearance that becomes rugose and opaque instead of smooth and translucent in the mutant, with the complementation strain becoming S/Tr again (Fig 2A) indicating that the phenotype is due to the lack of Hfq and not to a polar effect of the deletion and/or secondary mutations in the mutant. The colony morphology of *Vibrio* species often depends on the amount and the nature of extracellular polysaccharides produced by the cells [56, 57]. Opaque colony formation has been linked to an increase of expression of one of two EPS gene clusters in *V*. *cholerae* and *V*. *parahaemolyticus* [56, 58], a cluster that is absent in *V*. *alginolyticus*. In *V*. *vulnificus*, another cluster, named *brp* was shown to be important for rugose colony appearance [44]. These findings underscore the complexity of the relationship between colony aspect and exopolysaccharides. In our case, the opaque/rugose mutant had globally less EPS than the WT. Although, no genes linked to EPS production or secretion were identified as significantly affected by the *hfq* deletion in our transcriptomic study (S2 Table), we could identify two cluster of EPS genes that were differentially expressed. In the first one, out of eleven genes, 4 were up regulated 2–4 fold in the mutant, whereas in the second one, 5 out of 10 genes were down regulated 2–4 fold (data not shown). However, these genes were not included in our list in S2 Table because of an *FDR* higher than our cutoff of 0.001. Clearly, more studies will be required to understand the molecular basis of colony appearance in *V*. *alginolyticus*.

Deficiency in carbon and amino acids uptake and utilization caused by loss of *hfq* were extensively reported in both gram-positive and gram-negative bacteria [35, 52–54]. In this study, we found that a *V*. *alginolyticus hfq* mutant displayed several growth defects compared to the WT strain: in rich medium, it displayed an increased lag phase and reached a lower OD_600_ (S2 Fig) possibly reflecting a reduction in growth yield. Such reduction was also observed when the cells were growing on PTS sugars such as glucose, trehalose, fructose or N-acetyl glucosamine, as well as non-PTS sugars such as maltose (S2 Fig). Consistent with such phenotypes, we found that genes encoding PTS sugar transporter component II and genes involved in maltose transport and utilization had decreased expression. The mutant was also less efficient in using TCA cycle intermediates as carbon sources (S2 Fig) and, at the same time, a large portion of genes involved in glycolysis, TCA cycle and electron transfer chain had increased expression (S2 Table) in contrast to prior study in other bacterial species [54, 59]. The *hfq* mutant was also less able than the WT to grow on a variety of amino acids, both polar and hydrophobic. Decreased expression of two broad-specificity amino-acid ABC transporters (S2 Table) could contribute to these phenotypes. Finally, the mutant has increased biosynthesis of branched amino acids and glutamate, suggesting an accumulation of glutamate in the mutant. Reduction of the glutamate efflux by the homologues of the Aap transporter would also contribute to such accumulation. All together, deletion of *hfq* led to a general decrease of *V*. *alginolyticus* capability to utilize externally provided compounds, and an increase of central metabolism.

In keeping with decreased motility, and despite the fact we could not observe any apparent change in flagellar structure (S1 Fig), as many as 11 motility and chemotaxis associated genes (including several methyl accepting chemotaxis proteins, S2 Table) had reduced expression in the mutant, suggesting that motility reduction is linked to a decrease in chemotactic signaling and energy production, rather than to a direct regulation of flagellum biogenesis, although *fliC* genes, which encode flagellin, the subunit of the flagellum, were also less expressed. Decreased motility and EPS production may in turn contribute to less biofilm formation [57, 60].

The *hfq* mutant also displayed increased sensitivity to vancomycin, tobramycin, kanamycin, neomycin and fosfomycin, amongst 25 tested antibiotics (Table 4). These antibiotics do not belong to the same family (3 are aminoglycosides, fosfomycin is a phosphonic acid, and vancomycin is a glycopeptide), they don't have the same targets neither the same entrance mechanism in the cell. Hence, it is unlikely that Hfq plays a role in the resistance to these antibiotics through a unique mechanism. However, one contributing factor could be the role of Hfq in controlling the abundance of OMPs. Hydrophilic antibiotics are thought to pass the outer membrane barrier through porins. The fact that a majority of OMPs were significantly up regulated in the *hfq* mutant could contribute to the increased sensitivity to at least some of these antibiotics [61, 62]. Such changes could also contribute to the observed ECS response, as attested by the induction of the ECS sigma factor RpoE, as well as member of its regulon, such as *degQ*, *rseA*, or *smpA* [63](S2 Table).

In *E*. *coli*, level of OMPs is regulated by many Hfq dependent sRNAs [64]. In *V*. *cholerae*, two sRNAs that regulate OMP expression have been identified, VrrA and MicX. VrrA is transcribed by the ECS sigma factor RpoE, and represses the production of OmpT in an Hfq dependent way [22]. MicX was shown to repress the *ompC* gene in an *hfq* independent manner, whereas the primary transcript of MicX is processed by RNaseE in an *hfq* dependent fashion to a shorter transcript [65]. However, we were not able to identify significant changes through our transcriptomic approach neither of VrrA nor MicX levels. Further studies will be required to determine if these sRNAs or others, not yet identified, play a role in the observed changes.

Another sigma factor which, in *E*. *coli*, is regulated by many sRNAs is the stationary phase and stress response sigma factor RpoS [66, 67]. Maybe surprisingly, *rpoS* expression was not affected in the mutant. One possible reason could be the absence of significant expression of *rpoS* in our conditions (early exponential phase) preventing the detection of a significant difference between the wild type and the mutant.

Finally, in vibrios, conserved Hfq dependent sRNAs named *qrr* (four to five copies according to species) are negative regulators at low cell density of *hapR*, which encodes the master regulator of the quorum sensing system [17]. As could be expected, *V*. *alginolyticus hapR* expression increased 2.6 fold in the mutant (S2 Table). This increase of HapR in exponential phase, possibly linked to the loss of regulation by Qrrs in the *hfq* mutant could also contribute to the observed decrease of EPS and biofilm formation since in vibrios, HapR is a negative regulator of these processes [68].

In summary, our study once again points at the importance of posttranscriptional regulations mediated by sRNAs with the assistance of the RNA chaperone Hfq for tuning a wide range of functions in bacterial cells [69]. Undoubtedly, numerous sRNAs remain to be discovered in *V*. *alginolyticus* to account for the pleiotropic phenotypes of the *hfq* mutant.

## Supporting Information

S1 Fig Observation of flagella by a Hitachi H-7650 TEM in ZJ-T, *Δhfq*-T and *hfq*+-T. Magnification; ZJ-T: 2 500 x, *Δhfq*-T: 4 000 x and *hfq*+-T: 2 500 x .tif file. Magnification was adjusted for each strain to make a single cell and its flagella fill out the field of vision.
(TIF)

S2 Fig Effect of *hfq* deletion on growth in rich medium and utilization of various carbon sources. ZJ-T, *Δhfq*-T and *hfq*+-T were cultured in triplicates in TSB or M63 minimal medium supplemented with 0.4% (w/v) of the indicated saccharide or TCA cycle intermediates as sole carbon source. (Squares: ZJ-T, circles: *Δhfq*-T, triangles: *hfq*+-T). Indicated values correspond to the mean of three measurements, and error bars to standard deviations from three biological replicates
(TIF)

S3 Fig Effect of *hfq* deletion on the utilization of various amino acids as both carbon and nitrogen source. ZJ-T, *Δhfq*-T and *hfq*^+^-T were cultured in triplicates in M63 minimal medium minus D-glucose and (NH_4_)_2_SO_4_ and supplemented with the indicated amino acid as both carbon and nitrogen source. (squares: ZJ-T, circles: *Δhfq*-T, triangles: *hfq*^+^-T). Indicated values correspond to the mean of three measurements, and error bars to standard deviations from three biological replicates
(TIF)

S1 Table Nucleotide sequences of primers used in this study (XLSX)

S2 Table *Vibrio alginolyticus* genes are up/down regulated (> = 2-fold) by the absence of Hfq (XLSX)

S3 Table Known trans-encoded regulatory sRNAs identified in the genome of *Vibrio alginolyticus* ZJ-T (XLSX)
