# Supplementary figures and images for: The RNA Chaperone Hfq Is Involved in Colony Morphology, Nutrient Utilization and Oxidative and Envelope Stress Response in Vibrio alginolyticus
Source: PLoS One. 2016 Sep 29;11(9):e0163689. doi: 10.1371/journal.pone.0163689 (PMC5042437; doi:10.1371/journal.pone.0163689)

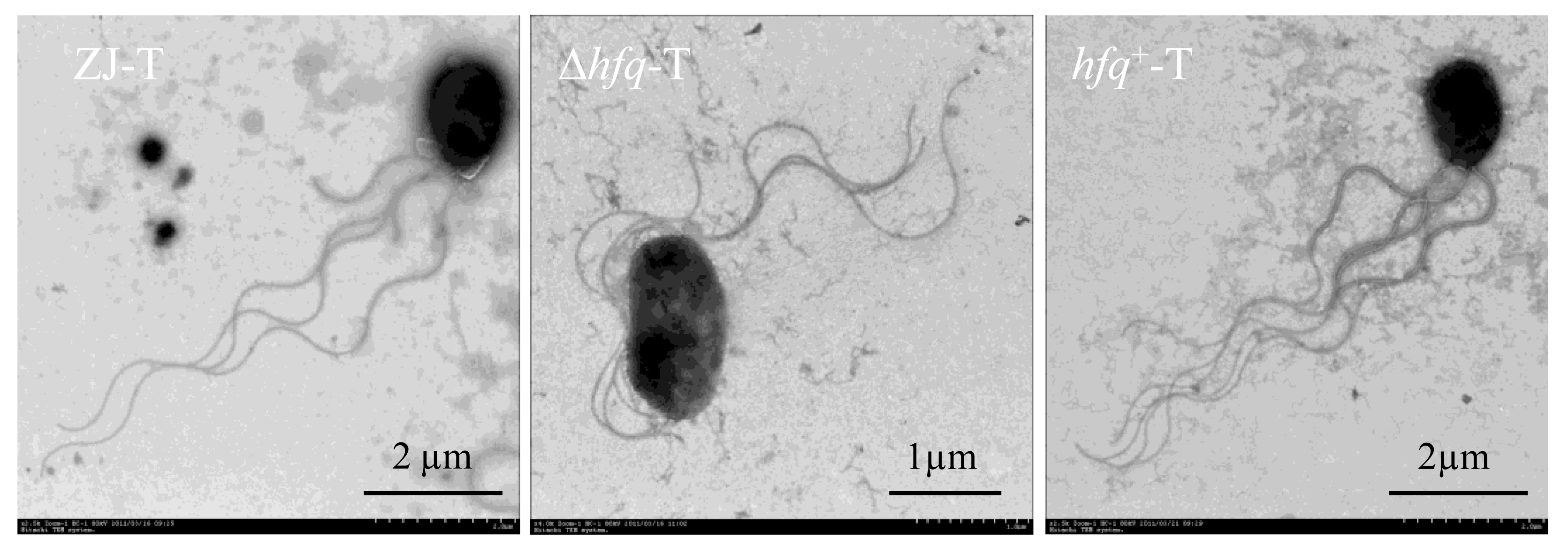

Supplement: S1 Fig — Magnification; ZJ-T: 2 500 x, Δhfq-T: 4 000 x and hfq+-T: 2 500 x .tif file. Magnification was adjusted for each strain to make a single cell and its flagella fill out the field of vision. (TIF) [file pone.0163689.s001.tif]

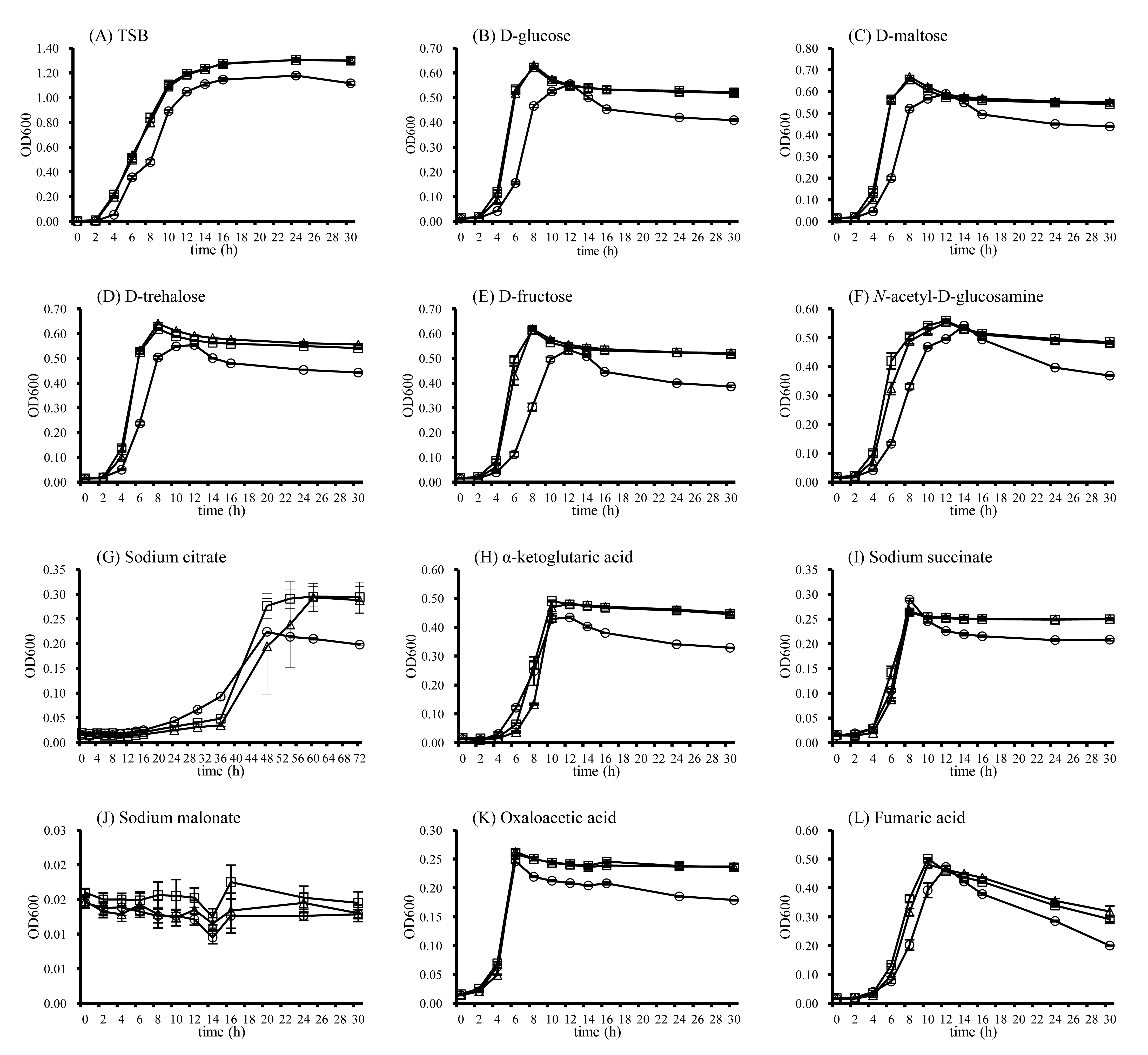

Supplement: S2 Fig — ZJ-T, Δhfq-T and hfq+-T were cultured in triplicates in TSB or M63 minimal medium supplemented with 0.4% (w/v) of the indicated saccharide or TCA cycle intermediates as sole carbon source. (Squares: ZJ-T, circles: Δhfq-T, triangles: hfq+-T). Indicated values correspond to the mean of three measurements, and error bars to standard deviations from three biological replicates (TIF) [file pone.0163689.s002.tif]

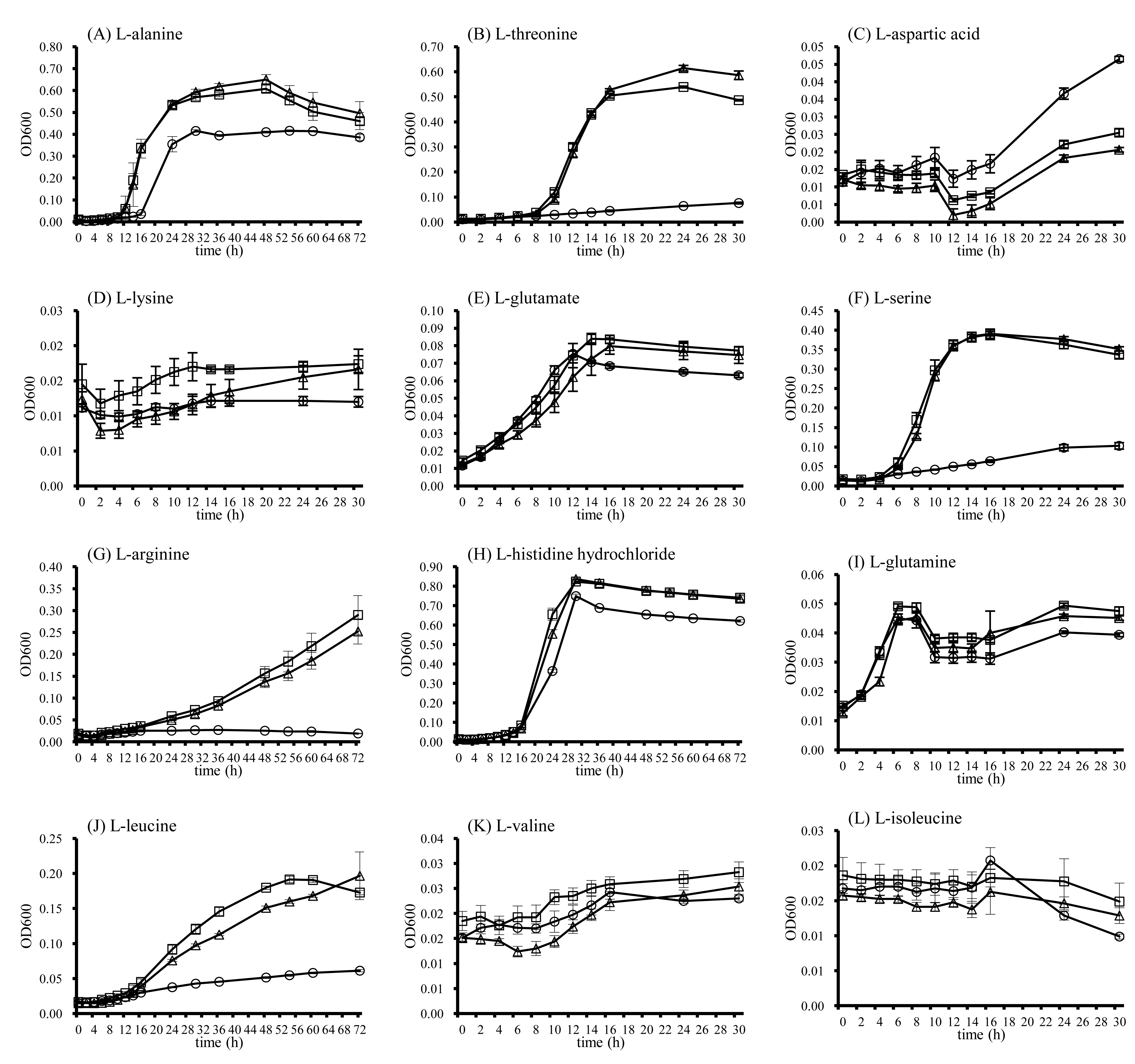

Supplement: S3 Fig — ZJ-T, Δhfq-T and hfq+-T were cultured in triplicates in M63 minimal medium minus D-glucose and (NH4)2SO4 and supplemented with the indicated amino acid as both carbon and nitrogen source. (squares: ZJ-T, circles: Δhfq-T, triangles: hfq+-T). Indicated values correspond to the mean of three measurements, and error bars to standard deviations from three biological replicates (TIF) [file pone.0163689.s003.tif]
